# Emerging Directions in Emotional Episodic Memory

**DOI:** 10.3389/fpsyg.2017.01867

**Published:** 2017-12-04

**Authors:** Florin Dolcos, Yuta Katsumi, Mathias Weymar, Matthew Moore, Takashi Tsukiura, Sanda Dolcos

**Affiliations:** ^1^Department of Psychology, University of Illinois at Urbana–Champaign, Champaign, IL, United States; ^2^Neuroscience Program, University of Illinois at Urbana–Champaign, Champaign, IL, United States; ^3^Beckman Institute for Advanced Science and Technology, University of Illinois at Urbana–Champaign, Champaign, IL, United States; ^4^Department of Psychology, University of Potsdam, Potsdam, Germany; ^5^Department of Cognitive and Behavioral Sciences, Graduate School of Human and Environmental Studies, Kyoto University, Kyoto, Japan

**Keywords:** emotion–cognition interactions, social cognition, emotion control, associative memory, individual differences (personality, sex, age), anxiety, depression, PTSD

## Abstract

Building upon the existing literature on emotional memory, the present review examines emerging evidence from brain imaging investigations regarding four research directions: *(1) Social Emotional Memory*, *(2) The Role of Emotion Regulation in the Impact of Emotion on Memory*, *(3) The Impact of Emotion on Associative or Relational Memory*, and (4) *The Role of Individual Differences in Emotional Memory*. Across these four domains, available evidence demonstrates that emotion- and memory-related medial temporal lobe brain regions (amygdala and hippocampus, respectively), together with prefrontal cortical regions, play a pivotal role during both encoding and retrieval of emotional episodic memories. This evidence sheds light on the neural mechanisms of emotional memories in healthy functioning, and has important implications for understanding clinical conditions that are associated with negative affective biases in encoding and retrieving emotional memories.

## Introduction

Research using functional brain imaging techniques in humans has established that the impact of emotion on cognition is subserved by complex interactions of functional networks and systems involved in various processes, which include basic emotion processing, perception, memory, and cognitive control (reviewed in Phelps, 2004; Phelps and LeDoux, 2005; Dolcos and Denkova, 2008; Dolcos et al., 2011, 2012, 2013; Murty et al., 2011; Murray et al., 2013; Dolcos and Denkova, 2015). Building upon the extant literature, the present review focuses on evidence regarding the following four emerging research directions in the field, regarding the impact of emotion on episodic memory: *(1) Social Emotional Memory*, *(2) The Role of emotion regulation in the Impact of Emotion on Memory*, *(3) The Impact of Emotion on Associative or Relational Memory*, and *(4) The Role of Individual Differences in Emotional Memory*. This review focuses on evidence identified from functional neuroimaging studies in healthy humans investigating the role of the amygdala (AMY) and its interaction with memory-related medial temporal lobe (MTL) brain regions, as well as the role of other brain regions (e.g., prefrontal cortex, PFC), during both encoding and retrieval of long-term emotional episodic memories^1^. Following a brief review of basic evidence concerning the neural mechanisms of emotional memory in humans, we will discuss in detail new evidence from the literature circumscribed by the four emerging topics mentioned above. We will end this review with a brief presentation of open issues to be clarified in future investigations.

## Neural Correlates of Emotional Episodic Memory: Basic Findings

Previous research has investigated the beneficial impact of emotion on episodic memory at various stages, from the initial encoding and early consolidation to subsequent retrieval of memory representations (reviewed in LaBar and Cabeza, 2006; Dolcos et al., 2012). This research typically considers two orthogonal dimensions of basic emotional properties, *arousal* and *valence* (Russell, 1980; Lang et al., 1993). In addition, to minimize possible confounds related to general perceptual processing and to isolate memory-related neural mechanisms, brain imaging studies of emotional episodic memory typically calculate the so-called subsequent memory effect – that is, differential brain activity associated with remembered vs. forgotten items – which is also called the Dm effect (difference due to memory) (Paller and Wagner, 2002; Prince et al., 2005; Shafer et al., 2011; Dolcos et al., 2012, 2017; Dolcos and Denkova, 2014). In general, current research mainly highlights the role of two mechanisms involved in the enhancing effect of emotion on episodic memory – (1) MTL-based [involving the AMY and memory-related MTL regions, such as the hippocampus (HC)] and (2) non-MTL-based mechanisms, the latter of which typically involves the PFC, among other regions (e.g., parietal cortex) (LaBar and Cabeza, 2006; Dolcos et al., 2012). The MTL mechanism contributes to the memory-enhancing effect of emotion through direct/bottom-up neurohormonal interactions between the AMY and memory-related MTL regions, during encoding (e.g., Dolcos et al., 2004b; Kensinger and Corkin, 2004; Kensinger and Schacter, 2006a; Sergerie et al., 2006; Ritchey et al., 2008), consolidation (McGaugh, 2004; Ritchey et al., 2008; see also LeDoux, 2007), and retrieval (Dolcos et al., 2005; Kensinger and Schacter, 2005) of emotional memories. The non-MTL mechanism contributes to the memory-enhancing effect of emotion through indirect/top-down interactions, by enhancing executive, attentional, and semantic processes (LaBar and Cabeza, 2006; Dolcos and Denkova, 2008).

### Emotional Memory Encoding

Previous neuroimaging studies have identified the critical role of the interaction between the AMY and the memory-related MTL brain areas, such as the HC and associated parahippocampal cortical regions, in successful encoding of emotionally arousing information (e.g., Hamann et al., 1999; Canli et al., 2000; Kilpatrick and Cahill, 2003; Dolcos et al., 2004b; Kensinger and Corkin, 2004; Richardson et al., 2004; Kensinger and Schacter, 2006a; Sergerie et al., 2006; Ritchey et al., 2008; see also Adolphs et al., 1997, 2000; Strange and Dolan, 2004 for related evidence from lesion/pharmacological studies). This research also showed that this within-MTL functional interaction is also important for the persistence of emotional memories over time (Ritchey et al., 2008). More recently, it has been shown that encoding of emotionally arousing stimuli over an extended period (>20 min) also induces a sustained arousal-related brain state, which overall contributes to greater recollection of unrelated neutral items encoded minutes following the initial encoding of emotional ones (Tambini et al., 2017). This suggests that transient exposure to emotional arousal can also create persistent “carry-over” effects, resulting in similar memory-enhancing effects for subsequently encountered neutral items (see also Lewis et al., 2005; Henckens et al., 2009; Fitzgerald et al., 2011; Joëls et al., 2011; Hermans et al., 2014).

Whereas AMY-MTL mechanisms are modulated primarily by arousal, the involvement of the PFC in emotional memory encoding seems to be influenced by valence (Dolcos et al., 2004a; Kensinger, 2004; Kensinger and Schacter, 2006b). For instance, there is evidence that AMY-HC functional coupling tends to be enhanced during encoding of negative stimuli, whereas PFC-HC coupling is stronger for encoding of positive stimuli (Ritchey et al., 2011; see also Mickley Steinmetz et al., 2010). In addition, there is also evidence showing that successful encoding of positive stimuli is associated with activation in frontal and parietal regions, whereas that of negative stimuli is associated with activation in temporal and occipital regions (Kensinger and Schacter, 2008; Mickley Steinmetz and Kensinger, 2009). This evidence lends support to other studies showing that encoding of positive information is related to activity in specific regions within the PFC (Dolcos et al., 2004a; Botzung et al., 2010a), likely due to increased processing requiring more cognitive resources (Poldrack et al., 1999; D’Esposito et al., 2000; Anderson et al., 2004), whereas encoding of negative information is related to temporal and occipital areas (Mickley and Kensinger, 2008), likely due to enhanced sensorial processing (Vuilleumier et al., 2004).

### Emotional Memory Retrieval

Emotional memory retrieval has been mainly studied using two types of settings. On the one hand, *laboratory micro-events*, such as a series of words or pictures, are encoded in laboratory settings and retrieved at relatively shorter intervals following encoding (e.g., from minutes to months). On the other hand, *autobiographical events*, referring to episodes from one’s personal past, are encoded in everyday life and may be retrieved after much longer intervals (e.g., years, decades). Similar to emotional encoding, previous studies provided evidence showing that successful emotional retrieval of laboratory micro-events involves the AMY-MTL mechanisms (Sharot et al., 2004; Dolcos et al., 2005; Kensinger and Schacter, 2005; Sergerie et al., 2006; see also Buchanan, 2007). The AMY also seems to be involved in successful retrieval of emotionally arousing and personally relevant autobiographical memories (AMs), following shorter retention intervals (Botzung et al., 2010b; see also Sharot et al., 2007a; Muscatell et al., 2010). In addition, AMY’s engagement during retrieval of more remote AMs seems to be dependent on task instructions associated with different levels of effortful processing (Smith et al., 2006), which may account for inconsistent findings regarding the AMY involvement in emotional AM retrieval (e.g., Markowitsch et al., 2000; Vandekerckhove et al., 2005). Because the level of effortful processing is increased when remembering temporally dispersed past events, this could lead to diversion of attentional resources from the emotional value associated with recollection of such events (Phan et al., 2002). This idea has been supported by evidence from a recent study in our group showing that attentional focus on emotional details (as opposed to non-emotional contextual details) during recollection of emotional AMs was associated with increased activity in the left AMY (Denkova et al., 2013b; see also Lieberman et al., 2007; Shafer et al., 2012 for similar effects of task goals and demands). Also similar to the evidence from encoding, AMY-MTL interactions seem to play a critical role in emotional retrieval, suggesting that the AMY and the memory-related MTL regions constitute a synergistic mechanism in which emotion and recollection enhance each other (Dolcos et al., 2005; Greenberg et al., 2005).

Importantly, retrieval-related functional interaction within the MTL also seems to be influenced by the PFC involvement. For instance, both increased AMY-HC connectivity and increased medial PFC activity have been observed during emotional memory retrieval (Smith et al., 2006). Moreover, the medial PFC also modulates AMY and HC activity during retrieval of emotional information, which suggests that activity in these regions can be modulated by task goals (Cunningham et al., 2008). This research also points the involvement of top-down processes linked to emotional memory retrieval, subserved by the medial PFC (see also Denkova et al., 2015). Finally, studies of memory retrieval for emotional laboratory (Maratos et al., 2001; Smith et al., 2004b, 2006; Erk et al., 2005) and autobiographical (Markowitsch et al., 2003; Piefke et al., 2003; Botzung et al., 2010b) events have also identified the involvement of PFC regions linked to processing of emotional valence. In particular, increased medial and orbital PFC activity was identified during retrieval of positive contextual information (Erk et al., 2005) and of positive AMs (Markowitsch et al., 2003; Piefke et al., 2003). In addition, increased activity in the lateral PFC was associated with recollection of positive (but not negative) AMs while focusing on emotional details compared to non-emotional ones (Denkova et al., 2013a). The medial orbital PFC has been associated with affective valuation, reward-related processing, and self-referential processing (Heinzel and Northoff, 2009; Roy et al., 2012; Delgado et al., 2016), whereas the lateral PFC, despite the functional heterogeneity of this region (e.g., Petrides and Pandya, 2002), has also been linked to the subjective experience of emotion (Barrett et al., 2007; Wager et al., 2008). Taken together, these findings suggest that the involvement of specific medial/orbital and lateral PFC regions during retrieval of positive emotional information may reflect processing of self-relevant positive/rewarding experience (see also Tsukiura and Cabeza, 2008).

In sum, the evidence discussed above supports the notion that the memory-enhancing effect of emotion during encoding and retrieval is linked to increased activity in and interaction between MTL and non-MTL areas, involved in emotion, mnemonic, and other types of processing. While the MTL-based memory mechanism is relatively more involved in arousal-dependent effects, valence-related effects are linked to connectivity of these regions within and outside the MTL. Regarding the role of non-MTL regions, involving the PFC and other cortical areas, available evidence suggests that its involvement during encoding and retrieval of emotional memories is relatively more sensitive to processing of valence and reflects higher order processes (e.g., semantic and working memory, attention, cognitive control, and self-referential processing).

## Emerging Directions in Emotional Episodic Memory

### Social Emotional Memory

Navigation of the complex social world requires knowledge acquired through previous social interactions to guide our adaptive behavior in various situations (Spreng and Mar, 2012; Tsukiura, 2012; Ciaramelli et al., 2013; Spreng and Andrews-Hanna, 2015). Thus, elucidating the complex interaction between emotion and social cognition is important, because of its implications for understanding the mechanisms underlying emotional memory for more ecologically valid situations than typical laboratory tasks. The relation between memory and social cognition has been previously identified (reviewed in Laurita and Spreng, 2017). For instance, there is evidence that trait empathy is positively associated with enhanced recognition memory performance in healthy individuals (Wagner et al., 2015), and is significantly reduced in patients with hippocampal amnesia showing declarative memory impairments (Beadle et al., 2013). These findings suggest an important link between long-term memory and social behavior, possibly mediated by healthy hippocampal functioning. Moreover, recent neuroimaging evidence points to the existence of a network of brain regions subserving memory processes for stimuli with *social relevance*, such as people’s faces and impressions formed based on them (e.g., Gilron and Gutchess, 2012; Tsukiura, 2012).

The extant evidence suggests that processing of stimuli with increased social relevance engages more effortful elaboration, and that memory for such stimuli is enhanced only when sufficient processing resources are available (Sakaki et al., 2012). More specifically, whereas biologically emotional stimuli are processed relatively more automatically by the involvement of and interaction between the AMY and the visual cortex, memory for socially emotional stimuli may also depend on more elaborative processes involving the interaction between the AMY and the medial PFC (Sakaki et al., 2012). Consistent with this idea, other investigations of social emotional memory have highlighted the involvement of the AMY and the medial/orbital PFC in a range of complex social cognitive functions, including detecting relevant social cues in the external environment, monitoring and interpreting internal emotional reactions, or processing subjective valuation of stimuli (Somerville et al., 2006; Harvey et al., 2007; Botzung et al., 2010a; Gilron and Gutchess, 2012; Tsukiura, 2012; Yaoi et al., 2015).

#### Encoding of Social Emotional Memories

Available evidence suggests that the AMY is involved not only in successful encoding of basic emotional stimuli, but also in that of stimuli with social and personal relevance (Harvey et al., 2007; Kleinhans et al., 2007; Botzung et al., 2010a; Tsukiura, 2012; see also Adolphs, 2010; Cunningham and Brosch, 2012). For instance, among a sample of dedicated basketball fans, AMY activity was preferentially involved in successful encoding of highly emotional memories with personal significance, especially those regarding positively valenced plays (e.g., a player from the fan’s team making a shot; Botzung et al., 2010a). Interestingly, increased activity in the AMY was also identified during imagination of positive future events compared to negative ones (Sharot et al., 2007b; Sharot, 2011). This evidence suggests that basketball fans may be more likely to consider positive plays as more personally significant than negative ones, and thus this enhanced self-relevance could lead to increased AMY activity linked to encoding of positive personal events (but see Northoff et al., 2009). Moreover, there is also evidence suggesting that the AMY is involved in encoding social information (e.g., faces, impressions), regardless of its valence (Said et al., 2009; Schiller et al., 2009; Vrtička et al., 2012). Because of its intrinsic motivational value and importance for survival, it is possible that social relevance in the environment is detected by the AMY, irrespective of basic emotional properties, such as valence and arousal (Harvey et al., 2007; Vrtička et al., 2012). These findings are consistent with evidence identifying a more general involvement of the AMY in social cognition and behavior, in tracking the subjective significance or relevance in the environment based on the current goals (Adolphs, 2010; Cunningham and Brosch, 2012).

Besides the AMY, the involvement of medial/orbital PFC regions has also been implicated in encoding social relevance (Harvey et al., 2007; Vrtička et al., 2012; Gutchess et al., 2015; see also Meyer and Lieberman, 2012; Meyer et al., 2015). For example, increased activity in the medial PFC was related to enhanced memory for the information encoded with reference to oneself – i.e., the self-reference effect (Macrae et al., 2004; Yaoi et al., 2015) – and also to encoding of impressions of other people based on face-behavior associations (Mitchell et al., 2004; Gilron and Gutchess, 2012; Cassidy et al., 2013). In addition, activity in the medial orbitofrontal cortex (OFC) was related to encoding of faces signaling positive social cues (Tsukiura and Cabeza, 2008, 2011a,b). These findings are consistent with the role of medial PFC regions in representations of one’s own and others’ minds (Northoff et al., 2006; Wagner et al., 2012), as well as in encoding and integration of the subjective value of stimuli (Delgado et al., 2016). Moreover, increased activity in the medial OFC, along with its increased functional connectivity with the HC, have been observed during successful encoding of socially rewarding stimuli (i.e., smiling and attractive faces) (Tsukiura and Cabeza, 2008, 2011a). Conversely, encoding of socially negative stimuli (e.g., untrustworthy or unattractive faces) was mediated by increased activity in and connectivity between the Insula and the HC (see also Botzung et al., 2010a; Tsukiura et al., 2013). Finally, activity in the anterior temporal lobe (ATL) structures along with the ATL connectivity with the AMY, MTL, and medial PFC regions have been implicated in social emotional memory encoding, with the ATL playing a role in representing and storing person identity information (Tsukiura et al., 2010; Olson et al., 2013; Collins and Olson, 2014; Spreng and Andrews-Hanna, 2015).

#### Retrieval of Social Emotional Memories

Similar to the evidence regarding encoding, retrieval of social emotional memories has been linked to the involvement of the AMY, HC, and PFC. Increased AMY activity was associated with retrieval of faces that had previously been encoded with emotional descriptions of behaviors (Somerville et al., 2006), and with social fairness learned in the context of an economic game (Singer et al., 2004). Furthermore, increased activity in, and interactions between, the AMY and the HC have been associated with false recollection of episodes resulting from conformity to others’ behavior (Edelson et al., 2011), thus suggesting that social interaction could lead to long-lasting memory alterations through the engagement of AMY-HC mechanisms. Regarding the PFC involvement, the right medial PFC seems to mediate retrieval of information related to social contexts, while the left medial PFC seems to underlie retrieval of self-generated information (Mano et al., 2011), thus suggesting lateralization of social vs. self-referential processing during episodic memory retrieval within the right and left mPFC, respectively. Finally, also similar to encoding, increased activity in the ATL and its increased functional connectivity with the HC have been linked to retrieval of person identity information (Tsukiura et al., 2008, 2011; Collins and Olson, 2014).

#### Functional Neural Systems and Networks in Social Emotional Memories

**FIGURE 1:**
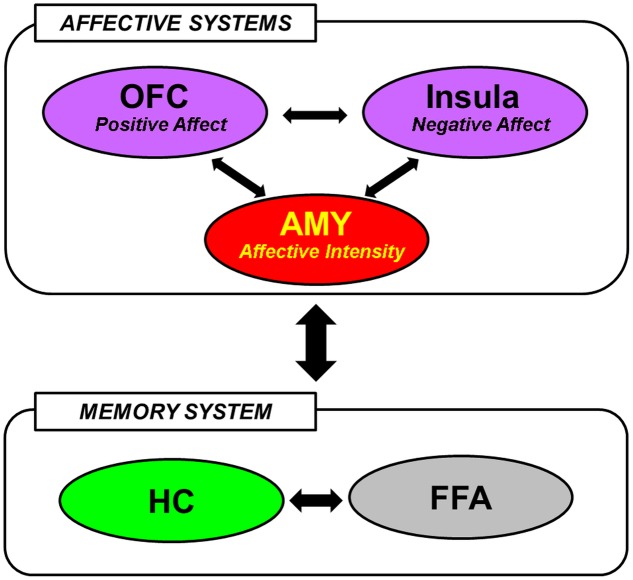
A hypothetical model of the neural mechanisms underlying the effect of face-based affective signals on memory for faces. AMY, amygdala; OFC, orbitofrontal cortex; HC, hippocampus; FFA, fusiform face area. From Tsukiura (2012), with permission.

The available evidence reviewed so far in this section suggests that encoding and retrieval of social information are associated with the involvement of brain regions typically implicated in basic emotion processing, social cognition, and memory. In integrating the differential contributions of these regions to the mechanisms underlying social emotional memory, Tsukiura (2012) has recently posited that the effect of emotional information on memory for faces may be mediated by interactions between *affective* (AMY, medial OFC, and Insula) and *memory* [HC, fusiform face area (FFA)] systems (**Figure 1**). In particular, the AMY detects general emotional properties of the faces, and interacts with the medial OFC and the Insula to process positive and negative signals from them, respectively. These regions of the affective system then modulate activity in the HC and the FFA, which have previously been implicated in encoding and retrieval of faces in general (Prince et al., 2009), to enhance memory for faces with affective information (Tsukiura, 2012). This framework is consistent with the notion that emotional memory processes are subserved both by bottom-up (e.g., AMY-HC) and top-down (e.g., MTL-PFC) mechanisms (LaBar and Cabeza, 2006; Dolcos et al., 2012), and also with an emerging account that the AMY serves as a functional hub across large-scale functional networks involved in various facets of social cognition and behavior (Bickart et al., 2014).

Moreover, evidence from recent studies examining large-scale functional networks of the brain suggests that complex processes involved in social emotional memory may be subserved by contributions from a network of regions whose activity represents a “default state” of the brain. These studies identified substantial spatial overlap between regions typically activated in tasks involving emotion processing, social cognition, and episodic memory, and those consistently activated when individuals are *at rest* – i.e., the *default-mode network* (DMN) (Schilbach et al., 2012; Spreng and Mar, 2012; Li et al., 2014). For instance, a recent meta-analysis identified the common involvement of the cortical midline structures [i.e., the dorsal subregion of the medial PFC (dmPFC) and precuneus] across studies examining emotion processing, social cognition, and the DMN, thus suggesting that introspective processes may be a common denominator across the three domains (Schilbach et al., 2012). Furthermore, extending this evidence to the memory domain, a recent study revealed that successful encoding of others’ faces in a self-referential manner (i.e., by imagining a potential friendship) was associated not only with increased activity in the (d)mPFC, but also with increased encoding-related functional connectivity between the cortical midline structures (i.e., the mPFC and precuneus) and HC (Yamawaki et al., 2017). Importantly, the dmPFC/precuneus and HC are considered part of the dorsomedial/core and medial temporal subsystems of the DMN, respectively (Spreng and Andrews-Hanna, 2015). Therefore, these findings suggest that complex memory encoding that involves self-referential processes in social context is associated with enhanced interactions among different functional subsystems within the DMN.

Finally, further expanding the traditional view of the DMN, an emerging view in the literature posits that the AMY, through its high intrinsic and task-evoked connectivity with some DMN regions, is part of a functional network involved in both maintenance of the brain’s default state and various socio-affective processes – i.e., the *extended social-affective default network* (Amft et al., 2015; see also Schilbach et al., 2008; Bickart et al., 2014). Taken together, emerging evidence suggests that social emotional memory is subserved by the complex interaction of functional systems/networks involved in a multitude of mental processes. A highly interactive nature of the neural mechanisms underlying social emotional memory reviewed here highlights the critical importance of examining measures of both neural activity and connectivity in future investigations on related topics.

Overall, recent functional neuroimaging evidence concerning the role of social information in emotional memory emphasizes the contributions of the AMY, HC, PFC, along with other regions involved in emotion processing and social cognition (e.g., Insula, ATL), to both encoding and retrieval of social emotional memories. The involvement of the AMY/HC and the medial PFC was linked to memory for positive stimuli with increased personal relevance, whereas the HC and Insula were linked to memory for negative social stimuli. Additionally, the AMY, medial PFC, and ATL were also associated with encoding and retrieval of social information, regardless of their valence, and these findings are consistent with evidence regarding the role of these regions in social cognition and behavior, in general. Emerging evidence from the investigations of large-scale functional networks has begun to reveal the relationships between the DMN and various social-affective and cognitive processes related to social emotional memory, with the midline cortical structures (dmPFC/precuneus) possibly serving as functional “hub” regions that integrate these processes. In sum, encoding and retrieval of social emotional information seem to involve complex interactions among both distinct and overlapping neural networks subserving basic emotion processing, social cognition, and memory processes, which may collectively allow us to integrate information from internal and external environments to adaptively navigate through the complex social landscape.

### The Role of Emotion Regulation in the Impact of Emotion on Memory

Research on emotion regulation (ER) has established that the ability to adaptively cope with emotionally challenging situations is vital for both physical and mental health. Although important progress has been made in elucidating the neural correlates associated with the effects of engaging specific ER strategies on *immediate* emotional experiences (e.g., Buhle et al., 2014; Dorfel et al., 2014), relatively less is known about the mechanisms linked to *long-term* consequences of engaging ER on emotional memory (but see Erk et al., 2010; Hayes et al., 2010; Binder et al., 2012; Denkova et al., 2013a,b, 2015). The two arguably most widely studied ER strategies in functional neuroimaging research are *cognitive reappraisal*, which involves attempts to change the meaning of stimuli/situations (e.g., by thinking that the situation is not real), and *expressive suppression*, which involves attempts to decrease emotionally expressive behavior (Gross, 2008). Available research suggests an advantage of reappraisal over suppression in reducing emotional experiences (Eippert et al., 2007; Kalokerinos et al., 2015; Olatunji et al., 2015), and dissociable neural mechanisms recruited by these ER strategies (Goldin et al., 2008; Hermann et al., 2014). More recently, ER research has also begun to elucidate the impact of other ER strategies involving attentional deployment [e.g., *focused attention (FA)*], which involves shifts in attention to or away from the emotional aspects of emotion eliciting stimuli or (memory for) events (Gross, 2008; Sheppes et al., 2014). The effectiveness of attentional deployment ER strategies has been confirmed by a recent meta-analysis (Webb et al., 2012), and the underlying neural mechanisms have been also investigated (McRae et al., 2010; Kanske et al., 2011; Denkova et al., 2013a,b, 2015; Dorfel et al., 2014).

Regarding the long-term effects of ER, several studies have provided evidence clarifying how the engagement of various ER strategies affects the impact of emotion on memory, at both behavioral (Richards and Gross, 1999, 2000; Dillon et al., 2007; Dunn et al., 2009; Kim and Hamann, 2012; Ahn et al., 2015), and neural (Hayes et al., 2010; Binder et al., 2012) levels. Clarification of the impact of ER on emotional memories has important implications for understanding and treating affective disorders, in which an excessive focus on distressing memories and emotional dysregulation are often among the core debilitating features (Nolen-Hoeksema et al., 2008; Williams and Moulds, 2008; Rubin et al., 2011; Cisler and Olatunji, 2012; Dolcos, 2013).

#### Emotion Regulation and Emotional Memory Encoding

Available evidence suggests that the engagement of cognitive reappraisal during memory encoding enhances subsequent memory for the reappraised stimuli (Richards and Gross, 2000; Dillon et al., 2007; Liu et al., 2015), even following extended intervals of 1–2 weeks (Kim and Hamann, 2012; Ahn et al., 2015), whereas the engagement of suppression tends to impair memory for the suppressed items (Richards and Gross, 1999, 2000; Dillon et al., 2007; Dunn et al., 2009). One potential explanation for the enhancing vs. impairing effects of reappraisal and suppression on subsequent memory, respectively, concerns the involvement of semantic elaboration involved in these processes. More specifically, it has been suggested that reappraisal requires significant stimulus elaboration, which leads to a deeper level of encoding of and better memory for the stimuli. However, suppression inhibits the emotional experience and/or expression, and therefore leads to impaired subsequent emotional memory (Dillon et al., 2007).

**FIGURE 2:**
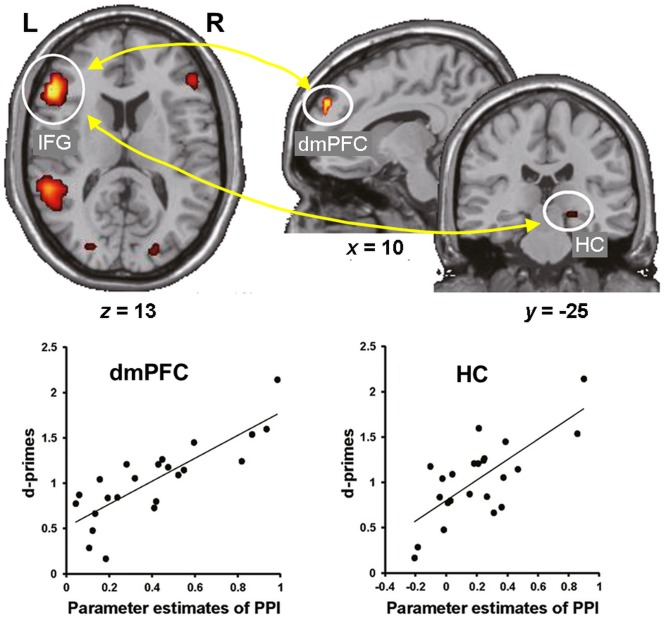
Functional interactions involved in the effect of emotional semantic elaboration on memory encoding. Functional connectivity between the left IFG and dmPFC as well as between the left IFG and right HC predicted subsequent memory for neutral items encoded via negative semantic elaboration. Similar patterns of brain-behavior associations were identified for positive semantic elaboration (not shown). IFG, inferior frontal gyrus; dmPFC, dorsomedial prefrontal cortex; HC, hippocampus. Adapted from Kaneda et al. (2017), with permission.

Consistent with these behavioral effects, evidence from neuroimaging studies points to dissociable neural engagement linked to successful encoding of reappraised vs. suppressed items (Hayes et al., 2010; Binder et al., 2012). In particular, the memory enhancement by reappraisal was associated with increased co-activation of the HC and left lateral PFC (inferior frontal gyrus, IFG) (Hayes et al., 2010), whereas the memory impairment by suppression was associated with decreased activity in the HC and its decreased connectivity with the lateral PFC (Binder et al., 2012). More recently, the idea of enhanced subsequent memory by semantic elaboration (and by accompanying self-generated emotions) has been extended to encoding of neutral stimuli. Specifically, subsequent memory for neutral stimuli was significantly enhanced when participants encoded the stimuli by imagining emotional background stories compared to passively viewing them (Kaneda et al., 2017). Furthermore, encoding of neutral stimuli via semantic elaboration, compared to passive viewing, was linked to increased activity in the left IFG and dmPFC, and functional connectivity between these regions as well as between the left IFG and right HC predicted subsequent memory for the neutral stimuli encoded via emotional semantic elaboration (**Figure 2**). These findings suggest that top-down emotion generation by semantic elaboration can enhance memory for perceptually neutral stimuli, and that this effect seems to be mediated by similar HC-PFC mechanisms involved in the impact of reappraisal on encoding of emotional stimuli. Overall, the available evidence regarding the impact of ER on emotional memory encoding demonstrates that the dissociable effects of reappraisal and suppression are linked to opposing patterns of interaction (increased vs. decreased, respectively) among brain regions involved in basic memory processes (HC) and in higher-order cognitive control and emotion processing (PFC).

#### Emotion Regulation and Emotional Memory Retrieval

##### Laboratory events

One of the most commonly studied ER strategies to inhibit retrieval of memories for laboratory events is *thought suppression* (Depue et al., 2006, 2007; Kupper et al., 2014; Benoit et al., 2015). Thought suppression is an attentional deployment strategy (Sheppes and Gross, 2012), which is typically studied using the so-called Think/No-Think paradigm during which participants first learn cue-target pairs associations, then are presented with the cues to suppress retrieval of the associated targets (Anderson and Green, 2001). In general, suppressing retrieval of unwanted memories has been linked to top-down influences of the lateral PFC on the HC (Gagnepain et al., 2014; Benoit et al., 2015), and this effect was stronger in participants who forgot more of the suppressed memories (Benoit and Anderson, 2012). More specifically, suppressing retrieval of emotional information has been associated with two-phase neural mechanisms, involving initial inhibition of visual regions (visual cortex) and subsequent inhibition of emotional memory regions (AMY and HC) by cognitive control regions (inferior and middle frontal gyrus, respectively) (Depue et al., 2007). Overall, this finding suggests that, whereas common mechanisms involving the PFC and HC underlie suppressing memory retrieval in general, specific patterns of neural activity are linked to suppressing emotional memory retrieval.

##### Autobiographical events

Investigation of the neural mechanisms underlying the impact of ER on autobiographical recollection has important implications for understanding both healthy functioning and affective disturbances. The few studies examining ER in the context of emotional AM retrieval have primarily focused on reappraisal (Kross et al., 2009; Fabiansson et al., 2012; Holland and Kensinger, 2013), and showed that the engagement of reappraisal can lead to reduced emotional experience associated with the recollected AMs. However, available evidence regarding the associated neural correlates so far is inconclusive, particularly with respect to the role of brain regions involved in emotion processing. For instance, down-regulation of emotional reactions via reappraisal during the (re)construction of AMs was associated with increased activity in the lateral and medial PFC; however, this was also associated with increased, but not decreased, activity in the AMY and Insula (Holland and Kensinger, 2013). In other studies, no specific activations related to reappraisal were identified, possibly due to the use of only a few unique memories that were repeated across different experimental conditions and/or of delayed instructions to regulate (i.e., after the engagement of memory retrieval) (Kross et al., 2009; Fabiansson et al., 2012; Holland and Kensinger, 2013).

**FIGURE 3:**
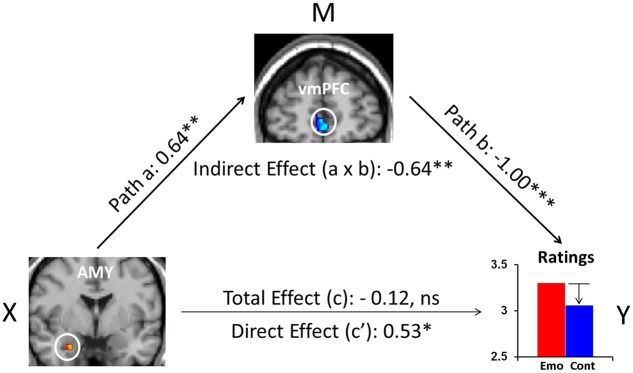
The mediating role of vmPFC during ‘distraction’ away from the emotional aspects of autobiographical recollections. Mediation analysis identified a significant (*p* = 0.009) negative mediation effect of vmPFC on the relation between the AMY and emotional ratings during focus on non-emotional aspects of memories, and a significant (*p* = 0.03) positive direct effect (path c′, X to Y controlling for M) between AMY and emotional ratings when controlling for vmPFC influence. Standardized coefficients and significance noted with asterisks are reported for each path. ^∗^*p* < 0.05, ^∗∗^*p* < 0.01, ^∗∗∗^*p* < 0.001 (two-tailed); ns, not significant. AMY, amygdala; vmPFC, ventromedial prefrontal cortex. From Denkova et al. (2015).

Recent evidence also points to the role of FA as an effective attentional deployment ER strategy during the recollection of emotional AMs (Denkova et al., 2013a,b, 2015). In particular, focusing attention on the non-emotional contextual aspects (e.g., time, location, other people present), and away from the emotional aspects of highly emotional personal memories, resulted in a decrease in self-reported emotional responses. This behavioral effect was accompanied by increased activity in the ventromedial PFC (vmPFC) and decreased activity in the AMY. Moreover, mediation analysis suggested a role of the vmPFC in integrating affective signals from the AMY and mediating their impact on the subjective re-experiencing of emotion, according to the current retrieval/attentional focus (**Figure 3**). While these findings refer to both pleasant and unpleasant AMs, valence-related differences were also identified in the PFC and MTL (Denkova et al., 2013a,b). Importantly, the finding regarding the role of the AMY described above (Denkova et al., 2015) challenges evidence from previous ER studies (Urry et al., 2006; Johnstone et al., 2007), mainly emphasizing top-down influences on AMY activity from the PFC regions involved in cognitive control, rather than reciprocal AMY-PFC influences, initiated in the AMY (see also Dolcos et al., 2006).

It is important to note that the manipulation used by Denkova et al. (2015) involves simply switching the retrieval focus during recollection of AMs. This remarkably subtle manipulation is consistent with evidence from other ER studies showing that manipulation of attention focus either on emotional or non-emotional aspects during working memory tasks can alter emotional responses (Thiruchselvam et al., 2011) and task performance (Iordan, 2016). Furthermore, this manipulation of retrieval focus can also be linked to emerging evidence regarding the effect of episodic specificity induction – i.e., brief training in recollecting episodic details (Madore and Schacter, 2016), which has revealed that focusing on very specific episodic details of recent events can enhance subsequent performance on a range of cognitive tasks (Madore et al., 2015; McFarland et al., 2017), and also increases psychological well-being (Jing et al., 2016). These findings are also consistent with recent evidence showing that training to recollect AMs with increased specificity may yield beneficial effects in depression (Raes et al., 2009; Watkins et al., 2009). The findings from the Denkova et al. (2015) study extend such investigations to emotional AMs, by showing that focusing on non-emotional aspects of AMs can influence the emotional (re)experience of such memories. These findings lend support to cognitive behavioral therapies involving ER training to “distract” from emotional aspects of personal memories, by focusing attention on their non-emotional contextual aspects, which in turn leads to reduced emotional experiences.

In sum, emerging evidence suggests that ER influences both encoding and retrieval of emotional memories, with different ER strategies associated with differential effects on subsequent memory (i.e., enhanced vs. impaired). More specifically, the engagement of cognitive reappraisal during encoding leads to enhanced emotional memory, whereas that of expressive suppression leads to impaired memory. Attentional deployment strategies, such as FA, seem to have beneficial effects on retrieval of emotional personal memories, because they can enhance the emotional impact of positive memories and reduce the impact of negative ones. These behavioral effects are coupled with differential recruitment and connectivity among PFC (lateral and medial) and MTL (AMY and HC) regions. Overall, in the context of the two main mechanisms of emotional memory discussed in the first section of this review, available evidence suggests that ER influences the bottom-up MTL-based mechanisms through the engagement of the top-down PFC-based mechanisms, which may also contribute to the memory-enhancing effect of emotions.

### The Impact of Emotion on Associative or Relational Memory

One important aspect of human memory concerns the binding of contextual information (e.g., time, place, or associative cues) that constitutes many disparate features of a unified event (Davachi, 2006; Ranganath, 2010). For instance, following the involvement in a traffic accident, environmental cues that were associated with the event (e.g., the make of the car, the color of surrounding vehicles, the song being played on the radio when the collision had occurred) may reactivate strong emotional memories, upon exposure to such cues at a later time. Furthermore, such associative mechanisms seem to be compromised in some clinical conditions, such as posttraumatic stress disorder (PTSD), in which events associated with traumatizing contexts can involuntarily trigger vivid recollection of distressing memories in the form of intrusive thoughts, flashbacks, or nightmares (Flor and Nees, 2014; Wilker et al., 2014). Therefore, recent research has begun to examine the impact of emotion on memory for items as a part of, or in relation to, other items (Chiu et al., 2013). Extant evidence identifies both enhancing and impairing effects of emotion on memory binding, and as discussed below, these opposing effects have been interpreted in the context of different (but not mutually exclusive) views (Christianson, 1992; Kensinger, 2009; Mather and Sutherland, 2011; Chiu et al., 2013; Bisby et al., 2016).

#### Behavioral Evidence

It has been known for a long time from the attention literature that emotional arousal tends to narrow attention to central cues, at the expense of peripheral, irrelevant cues (Easterbrook, 1959). Such prioritization of resources toward central aspects of information can result in enhanced memory for the central aspects, compared to the peripheral or contextual aspects that are attended less (Burke et al., 1992; Christianson, 1992; Buchanan and Adolphs, 2002). Therefore, emotional arousal or salience seems to weaken the integration of central aspects with peripheral contextual information into a unified memory representation (Mather, 2007). This phenomenon has also been referred to as the *emotion-induced memory trade-off*, in which memory is enhanced for the central emotional content of a stimulus but impaired for the associated neutral contextual information (Kensinger, 2009).

Among the various accounts for these opposing effects of emotion on memory (Mather, 2007; Kensinger, 2009; Chiu et al., 2013), one suggests that emotional arousal has dissociable effects on memory binding depending on certain aspects related to an event. In particular, Mather (2007) proposed that, whereas emotion can enhance memory for *within*-object features, it impairs memory for *between*-object features, the latter of which do not take advantage of emotional arousal (see also Kensinger, 2009 for a similar distinction between the intrinsic vs. extrinsic information). These assumptions were derived from a number of studies using either within-item or between-item binding tasks (reviewed in Chiu et al., 2013). For instance, memory was enhanced for specific intrinsic features, such as color (Doerksen and Shimamura, 2001; D’Argembeau and Van der Linden, 2004) or location (Mather and Nesmith, 2008; Nashiro and Mather, 2011) of emotional stimuli, compared to the same features being associated with neutral stimuli. By contrast, memory was impaired for extrinsic features and contextual details of emotional stimuli, when emotional objects were accompanied by neutral scenes (Kensinger et al., 2007a), when neutral objects were superimposed on emotional scenes (Touryan et al., 2007b), or when emotional scenes were associated with a colored frame (Rimmele et al., 2011).

**FIGURE 4:**
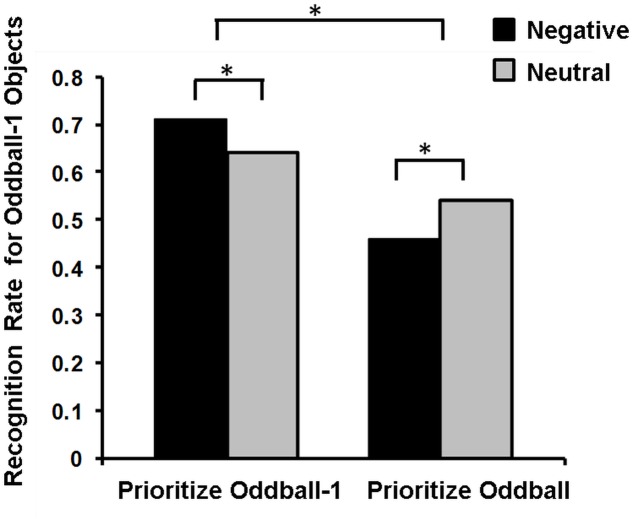
Memory for Oddball-1 objects in the Prioritize Oddball-1 and Prioritize Oddball Conditions. Negative compared to neutral images lead to better memory for the preceding neutral objects (oddball-1) when people prioritize these objects (Prioritize Oddball-1 Condition), but to worse memory when people do not prioritize these objects (Prioritize Oddball Condition). ^∗^Significant difference. Adapted from Sakaki et al. (2014).

Mather’s (2007) view regarding these opposing effects of emotion on memory has been recently postulated in the so-called *Arousal-Biased Competition (ABC) Theory* (Mather and Sutherland, 2011), which underscores the role of another important factor contributing to memory binding, namely, *attentional priority*. According to this account, emotional arousal enhances processing of stimuli with the highest priority (either due to bottom-up salience or top-down relevance), and impairs processing of those with lower priority (Mather and Sutherland, 2011). Thus, emotional arousal may enhance associative memory for features of high-priority items (e.g., color or location of an item) and impair memory for neutral items, when presented at the same time (or closely in time, see Sakaki et al., 2014) with emotional items. For instance, presentation of emotional stimuli enhanced or impaired memory for the preceding neutral stimuli, depending on whether these neutral stimuli were highly prioritized or not, respectively (**Figure 4**; Sakaki et al., 2014).

Importantly, the ABC theory also seems to account for previous behavioral evidence identifying enhanced memory for neutral words presented in the context of emotional words (Guillet and Arndt, 2009), or when neutral objects were placed on emotional scenes (Smith et al., 2004b, 2005). In general, memory should be impaired for neutral stimuli following an attention narrowing stimulus (Easterbrook, 1959) or a between-object feature (Mather, 2007). In these studies, participants were instructed to learn word-word associations (Guillet and Arndt, 2009) or to mentally integrate or connect objects and scenes during encoding (Smith et al., 2004b), which might have given such associations high “attentional priority,” resulting in facilitation of binding between emotional and neutral information. Taken together, these findings illustrate both enhancing and impairing effects of emotion on memory binding, and that these opposing effects can be explained by differences in attentional deployment toward, and prioritization of, emotional items and the associated features (Easterbrook, 1959; Mather, 2007; Kensinger, 2009; Mather and Sutherland, 2011).

#### Brain Imaging Evidence

##### Encoding

Current evidence discussed in this review so far suggests that the effects of emotion on memory binding are mediated by the AMY and its modulation of activity in regions within the MTL that are important for relational representations (HC), such as the binding of item and contextual information (Davachi, 2006; Ranganath, 2010). However, available empirical evidence has been inconclusive, in part due to experimental designs examining different aspects related to an event (e.g., within vs. between features). For instance, memory was enhanced for emotional items compared to neutral ones presented in the context of neutral backgrounds, and this effect was associated with increased activity in the AMY, HC, and PFC regions (Waring and Kensinger, 2011). Similarly, increased AMY activity was identified for subsequently remembered emotional words compared to neutral words (Kensinger and Schacter, 2006a; Dougal et al., 2007). Such memory-enhancing effects, as reflected in greater response in AMY and other MTL regions, however, were not observed for emotional stimuli when the associated contextual details (i.e., semantic judgment or color) were tested (Kensinger and Schacter, 2006a; Dougal et al., 2007). This finding is consistent with evidence regarding the role of the AMY in emotional gist memory (Adolphs et al., 2005). In contrast, increased activity in the HC was related to correct memory of contextual details (Ranganath, 2010), for both emotional and neutral stimuli (Kensinger and Schacter, 2006a).

Together, these findings support the idea that the AMY is specifically involved in enhanced memory for aspects that are intrinsically linked to an emotional event (see also Thoresen et al., 2012), but not in memory for its extrinsic aspects. However, this prioritized processing of emotion-associated information, which could lead to overall enhanced subsequent memory, is not limited to intrinsic aspects of the emotional event. More specifically, available evidence suggests that instructions to intentionally connect objects and their locations with emotional or neutral background scenes can lead to enhanced memory for the objects’ location when presented with emotional scenes, and this effect was associated with increased AMY activity during encoding (Luck et al., 2014). However, it remains unclear whether or not such memory-enhancing effect of emotion is related to preferential processing based on prioritization, as suggested by Mather and Sutherland (2011). Extant evidence also identifies alterations in functional coupling between the AMY and visual processing regions during encoding, depending on whether information is prioritized or not (Lee et al., 2014). This suggests that emotional arousal can indeed amplify visual processing of stimuli with high priority, which might influence subsequent MTL-based binding into a long-lasting memory representation. This idea is consistent with electrophysiological evidence identifying early enhanced perceptual and elaborative processing in conditioning studies using aversive electrical shocks (for review see Miskovic and Keil, 2012) or emotional background pictures (Ventura-Bort et al., 2016a), as unconditioned stimuli.

##### Retrieval

Consistent with evidence from studies of emotional item memory (Dolcos et al., 2005), increased activity in the AMY, HC, and PFC was associated with recognition of words that have been encoded in the context of emotional sentences compared to those encoded in association with neutral ones (Maratos et al., 2001). Increased AMY activity during retrieval was also identified for correctly recognized negative scenes than forgotten ones, when cued by either an associated negative or neutral scene (Bisby et al., 2016). Moreover, increased retrieval-related activity was identified in the AMY, HC, and PFC when objects (and source information) from emotional background scenes were remembered (Smith et al., 2004b, 2005). Notably, successful retrieval of objects that have been associated with emotional scenes during encoding was associated with increased functional connectivity between the AMY and HC (Smith et al., 2006), suggesting that these regions support the retrieval of emotion-associated information from episodic memory when successfully integrated (see encoding instructions by Smith et al., 2004b, 2005, 2006; but also see Takashima et al., 2016 for HC involvement when emotional context memory is inaccurate).

**FIGURE 5:**
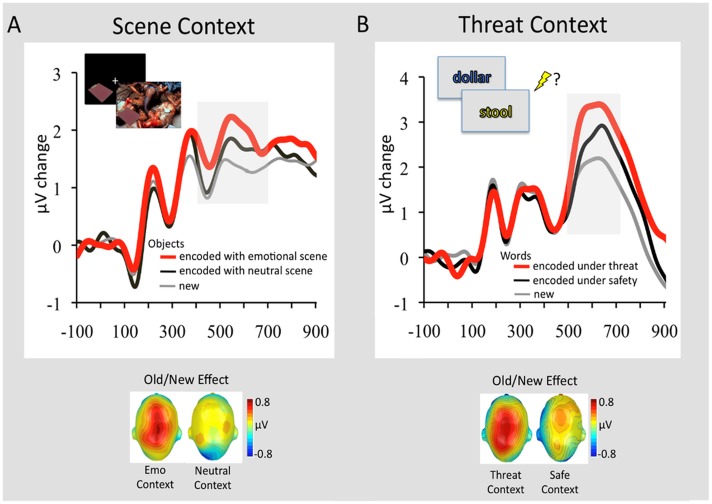
Retrieval-related ERP signature (Old/New Effect) of stimuli previously associated with emotional and neutral contexts. **(A)** Grand average ERP waveforms at a representative centro-parietal sensor cluster for correctly recognized objects that had been encoded in the context of an emotional background scene (red line) or neutral background scene (black line) and correctly classified new objects (gray line). In the upper left of the figure is displayed the encoding sequence of the experiment. In this experiment 144 objects were presented in the context of 144 background scenes (48 pleasant, 48 neutral, 48 unpleasant). Objects were presented first followed by the background scene, to avoid direct competition between emotional backgrounds and neutral objects. To facilitate memory binding, participants were instructed to imagine that the object is a part of the scene. The graph below illustrates the scalp topographies of the ERP difference (old minus new; 400–700 ms) separately for objects originally paired with emotional or neutral scenes. Adapted from Ventura-Bort et al. (2016b). **(B)** Grand average ERP waveforms at a representative centro-parietal sensor cluster for correctly remembered words encoded in a font color (see encoding sequence in the upper left) that signaled threat of shock (red line) or safety (black line) and correctly classified new words (gray line). The graph below illustrates the scalp topographies of the ERP difference (old minus new; 500–700 ms) separately for emotional words originally encoded under threat or safety. Adapted from Weymar et al. (2013), with permission.

These fMRI findings, identifying memory-enhancing effects of emotion on associative memory binding, are also supported by a recent study examining brain activity using event-related potentials (ERPs) (Ventura-Bort et al., 2016b). Using an experimental paradigm similar to those used by Smith et al. (2004a,b, 2005, 2006), this study identified larger parietal positivity during retrieval of neutral objects previously encoded with emotional, but not neutral, background scenes (**Figure 5A**; Ventura-Bort et al., 2016b). The observed parietal *Old/New effect* (>400 ms following the stimulus onset) has been associated with recollection-based remembering (Rugg and Curran, 2007; Weymar and Hamm, 2013), thus suggesting that objects from emotional contexts were better recollected than those from neutral contexts. A similar ERP signature of memory retrieval has been identified for stimuli encoded under threat of shock (Weymar et al., 2013, 2014). More specifically, subsequent recollection was enhanced for stimulus words encoded under a condition in which a painful electric shock was anticipated (signaled by the color of a stimulus – i.e., within-object binding), compared to those encoded under a safety (no-shock) condition (**Figure 5B**). Interestingly, the observed context effects on memory were most reliable for emotional events (Weymar et al., 2013, 2014), which fits with the ABC theory (Mather and Sutherland, 2011) positing that arousal (e.g., threat of shock) during encoding may facilitate subsequent recollection of prioritized information (e.g., emotionally salient words). These studies also show that a context bound to an event is not restricted to the actual experience but also to its mere anticipation (Maren et al., 2013).

Finally, the findings from Ventura-Bort et al. (2016b) point to another important factor that may have an impact on memory binding, namely, the *retention interval*. More specifically, enhanced ERP Old/New effects were observed for objects from emotional contexts when memory was tested 1 week after initial encoding of the stimuli, but not when it was tested immediately or 24 h following encoding (Smith et al., 2004a; Jaeger et al., 2009; Jaeger and Rugg, 2012). Several studies have shown that longer retention intervals facilitate consolidation processes, leading to memory enhancement for highly arousing emotional stimuli, compared to low arousing neutral ones (Dolcos et al., 2005, 2012, 2017; LaBar and Cabeza, 2006; Ritchey et al., 2008; Weymar et al., 2009, 2011; Weymar and Hamm, 2013; Yonelinas and Ritchey, 2015). Therefore, emotion may facilitate associative binding after longer delays, and this idea is also consistent with a recent study identifying similar advantages of longer vs. shorter delays for associative binding (Pierce and Kensinger, 2011). Hence, future research should also consider retention intervals as an important factor when examining the effects of emotion on associative binding.

#### An Emerging Account: The Role of “Unitization”

An alternative (but not mutually exclusive) explanation for the opposing effects of emotion on memory binding has been recently proposed by Chiu et al. (2013), who posit that the enhancing or impairing effects may be linked to whether or not items are represented in an *unitized* manner in memory. This view was derived from a growing body of literature pointing to distinct MTL regions supporting item (perirhinal cortex) vs. relational (parahippocampal cortex/HC) memory representations (Cohen et al., 1999; Davachi, 2006; Ranganath, 2010). Previous fMRI studies (Haskins et al., 2008; Staresina and Davachi, 2010) have shown that the perirhinal cortex may also contribute to simpler forms of associative learning based on unitization, which involves representation of separate components (e.g., an object and its color or location) as a single unit (Graf and Schacter, 1989). Therefore, memory for items and “unitized” items can be mediated by similar mechanisms (perirhinal cortex), unlike memory representations that involve more complex associations, for instance, in temporal, spatial, and/or situational domains, which rely on the HC (Konkel and Cohen, 2009). This idea is also supported by recent ERP evidence showing differential ERP Old/New effects related to familiarity vs. recollection for high-unitized and low-unitized associations (Diana et al., 2011).

Based on the extant evidence, Chiu et al. (2013) suggest that emotion leads to enhancement of “item-only” or unitized memory representations, but it impairs more complex HC-dependent relational representations. Therefore, emotion leads to memory enhancement in tasks where the nature of the item-source association is more intrinsic (e.g., color or location) and therefore allows a single representation; however, it impairs memory when complex HC-dependent relational memory representations are required, for instance, in tasks using object-background associations (Kensinger et al., 2007a) or item-pairs (Mather and Knight, 2008). Importantly, when separate objects or features receive equivalent attention, for instance, by instructions to mentally integrate or connect certain items (Smith et al., 2004a,b; Ventura-Bort et al., 2016b), this can facilitate unitized processing and subsequent remembering. There is evidence showing that such integrative mental imagery relies more on amygdalar and parietal processing, and less on frontal and hippocampal processing, thus suggesting that unitized emotional associations may be less mediated by HC-dependent mechanisms (Murray and Kensinger, 2014; see also Ventura-Bort et al., 2016b for discussion of the involvement of familiarity-based recognition and valence). More research is required, however, in order to substantiate this view, given that this idea has not been empirically tested using systematic manipulations of unitization.

Overall, the enhancing and impairing effects of emotion on memory binding that have been identified in the literature so far seem to be explained by differences in attentional deployment toward emotional items and associated features during encoding, whether information is processed in a unitized or complex manner, or differences related to consolidation processes (retention retrieval). The available evidence suggests that emotion may facilitate memory for contextual details or other surrounding stimuli when viewed as intrinsic or unitized to the emotional event itself, via the involvement of the AMY and its interaction with perceptual regions (see also Mather et al., 2015 for the possible role of the locus coeruleus) that promote memory binding in the MTL regions. Successful retrieval of emotion-associated contextual information seems to be associated with increased activity in the AMY and memory-related MTL regions as well as enhanced electrocortical activity related to recollection.

### The Role of Individual Differences in Emotional Episodic Memory

Emerging research also highlights the importance of investigating how the neural mechanisms involved in emotional memory vary between individuals. Such investigations help to further elucidate the underlying brain mechanisms in both healthy and clinical groups^2^, including those impacted by affective disorders which are characterized by dysfunctional emotional memory (Nolen-Hoeksema et al., 2008; Dolcos, 2013). It is therefore crucial to clarify the factors that might give rise to individual differences in the contribution of emotion- and memory-related brain regions and their interaction with cognitive control regions to emotional memory. The present discussion focuses on *personality*, *sex*, and *age differences* (e.g., Drabant et al., 2009; Mak et al., 2009; Canli et al., 2002b; Domes et al., 2010; Mather, 2016; Katsumi et al., 2017).

#### Personality-Related Differences

**FIGURE 6:**
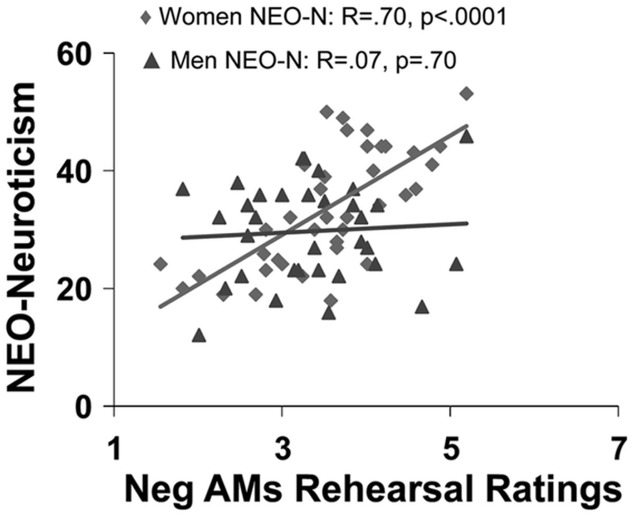
Sex differences in the effect of neuroticism on affective autobiographical retrieval and rehearsal. Neuroticism (NEO-N) predicts frequency of rehearsing negative AMs only in women. From Denkova et al. (2012), with permission.

Available evidence suggests that general personality traits, such as extraversion, neuroticism, and anxiety, as well as more specific traits indexing habitual engagement of ER, modulate the neural mechanisms underlying the impact of emotion across different processes, including perception, attention, emotional response, and memory (Canli et al., 2002b; Hamann and Canli, 2004; Touryan et al., 2007a; Hooker et al., 2008; Drabant et al., 2009; Eden et al., 2015). For example, extraversion has been linked to enhanced recall of positive memories (Mayo, 1983; Rusting, 1999) and more positive affective states following recall of positive AMs (Denkova et al., 2012). Extraversion has also been associated with a positive affective bias in memory, which in turn is associated with enhanced AMY engagement during memory encoding (Haas and Canli, 2008). In contrast, neuroticism is linked to enhanced recall of negative information (Bradley and Mogg, 1994), including negative personal memories, as shown by an increased frequency of rehearsing negative AMs in women (**Figure 6**) and greater proportion of recollecting negative AMs in men (Denkova et al., 2012). Furthermore, extant evidence suggests that neuroticism modulates the neural mechanisms that are altered by the impact of emotion on attention and memory (Ormel et al., 2013). The negative affective bias in emotional memory related to neuroticism might be due to increased activation of brain networks engaged during attentional processes, driven largely by greater recruitment of the AMY to negative information during memory encoding (Haas and Canli, 2008). Consistent with this idea, individuals with higher levels of neuroticism showed increased AMY and HC engagement when learning associations between fearful and neutral stimuli, which in turn was associated with enhanced memory for the neutral stimuli learned in association with the fearful stimuli (Hooker et al., 2008). Additionally, neuroticism was linked to reduced connectivity within the top-down executive network (Carballedo et al., 2015).

Trait anxiety is also linked to enhanced encoding and retrieval of negative stimuli (Russo et al., 2006) and modulates AM. Highly anxious individuals seem to have difficulties suppressing the effects of negative AMs, which can be observed in a decrease of the fading of emotional experience linked to recollection of negative personal memories over time (Walker et al., 2014). Furthermore, trait anxiety appears to modulate the neural mechanisms subserving emotional learning, including AMY’s engagement. For example, highly anxious individuals showed enhanced AMY response when exposed to items previously learned in association with negative emotional stimuli (Eden et al., 2015). Overall, this emerging evidence suggests that neuroticism and anxiety are associated with greater engagement of the bottom-up systems subserving emotional memory, leading to enhanced encoding and retrieval of negative associations. Additionally, trait anxiety is linked to reduced resting-state connectivity between the AMY and the dorsal attention network as well as to increased connectivity between the AMY and the ventral attention network (He et al., 2016). This evidence appears consistent with the attentional control theory (Eysenck et al., 2007), which proposes that anxiety modulates the dynamics between the top-down and bottom-up systems of attention in the brain.

Furthermore, available evidence suggests that habitual engagement of emotion control can also influence the effect of emotion on cognition. On the one hand, cognitive reappraisal, which is positively related to extraversion and negatively to neuroticism, is linked to more positive affective states and overall greater psychological well-being (Gross and John, 2003), as well as with a bias in remembering positive personal memories (Denkova et al., 2012). On the other hand, expressive suppression, which is negatively related to extraversion, is associated with increased negative affect and greater tendency toward anxiety symptoms (Llewellyn et al., 2013). Habitual engagement of suppression has also been associated with reduced confidence in memory accuracy and decreased experience of emotional and sensory details during autobiographical retrieval (Rubin and Seigler, 2004), as well as with increased recollection of negative AMs and enhanced negative affective states following retrieval of unpleasant memories in women (Denkova et al., 2012).

#### Sex Differences

As alluded to in the previous section, extant evidence points to sex differences in emotion processing (Stevens and Hamann, 2012; Andreano et al., 2014) and in emotional memory (Cahill, 2003; Hamann, 2005; Andreano and Cahill, 2009). For example, compared to men, women remember more emotional personal/AMs (Seidlitz and Diener, 1998; Davis, 1999), and show enhanced overall brain response during encoding of emotional memories (Canli et al., 2002a). Previous research on sex differences in the underlying mechanisms of emotional memory also highlights a hemispheric asymmetry in the recruitment of AMY, with the left AMY linked to successful emotional memory encoding in women, and the right AMY linked to successful encoding in men (Cahill et al., 2001; Canli et al., 2002a; but see Fischer et al., 2007). There is also evidence that consolidation of emotional memory is altered by sex differences (Mackiewicz et al., 2006), and that the sex-related lateralization in the AMY associated with emotional memory encoding appears to also be influenced by sex differences in the stimuli being encoded. More specifically, there is evidence pointing to preferential engagement of the left AMY during successful encoding of female emotional faces in women, and to preferential engagement of the right AMY during successful encoding of male emotional faces in men (Armony and Sergerie, 2007).

The idea that women preferentially process same-sex stimuli is consistent with findings that women tend to have increased preference for socially relevant stimuli, in general (e.g., faces and people as opposed to scenes) (Proverbio et al., 2008a,b). This has led to the idea that feminine and masculine roles as developed by society, rather than the sex *per se*, contribute to the observed differences in emotional memory (Cahill et al., 2004) between women and men. This interpretation is consistent with findings showing sex differences in AMY activity associated with opposing effects in memory for peripheral vs. central details. Namely, the engagement of the left and right AMY was associated with enhanced consolidation of peripheral vs. central aspects of emotional events in women and men, respectively (Cahill and van Stegeren, 2003). Furthermore, there is also evidence that those participants with greater feminine traits recalled more the peripheral features of emotional information, whereas those with greater masculine traits recalled more the central aspects (Cahill et al., 2004). Overall, these findings demonstrate that femininity is linked to relatively enhanced vs. impaired recall of peripheral vs. central aspects of emotional information, respectively, with evidence that the former involves recruitment of the left AMY.

Emerging evidence also shows that women recall fewer positive and more negative AMs than men do (Denkova et al., 2012), although women and men show similar assessment of phenomenological properties (e.g., vividness and arousal) of their personal emotional memories (Young et al., 2013). Furthermore, available evidence suggests that recall of emotional AMs and post-retrieval mood is differently influenced by habitual ER, in women and men (Denkova et al., 2012). That is, habitually engaging suppression as a strategy in women may be inefficient and could come at the cost of overall enhanced recollection of negative AMs and greater post-retrieval negative mood (Denkova et al., 2012). At the neural level, retrieval of emotional AMs has been shown to be related to common and dissociable brain activity in women and men. More specifically, although women and men showed similar activation of the PFC, AMY, and memory-related MTL regions during retrieval of emotional AMs, remembering negative AMs was associated with greater engagement of brain regions such as the AMY, whereas remembering positive AMs was associated with greater engagement of lateral PFC, in women compared to men (Young et al., 2013). Overall, the evidence regarding sex differences in emotional memory is consistent with the idea that women perceive, express, and experience emotions to a greater extent than men do (Briton and Hall, 1995; Meyers-Levy and Loken, 2015), and with evidence highlighting both advantages and disadvantages associated with sex differences in emotion processing, in general. While there is evidence in support of enhanced emotional competence in women (Hall and Matsumoto, 2004; Montagne et al., 2005; Collignon et al., 2010), there is also evidence pointing to women’s increased vulnerability to affective disturbances (Nolen-Hoeksema, 2001; Kessler, 2003; Bekker and van Mens-Verhulst, 2007).

#### Age-Related Differences

Healthy aging is characterized by both an overall preserved ability to process and remember emotional information (Phillips et al., 2002; Keightley et al., 2006; Mather, 2006; St. Jacques et al., 2009, 2010; Dolcos et al., 2014) and by an enhanced ability to spontaneously control emotion (Gross et al., 1997; Mather and Knight, 2005; Dolcos et al., 2014). These are reflected in the so-called age-related *positivity effect* – i.e., the tendency to enhance positive emotions and reduce negative emotions (Mather, 2016). Thus, examination of age-related differences in emotion processing and emotional memory also has the potential to help clarify the underlying mechanisms of emotional memory in mood and anxiety disorders, and to identify possible therapeutic interventions in these and other related disturbances.

**FIGURE 7:**
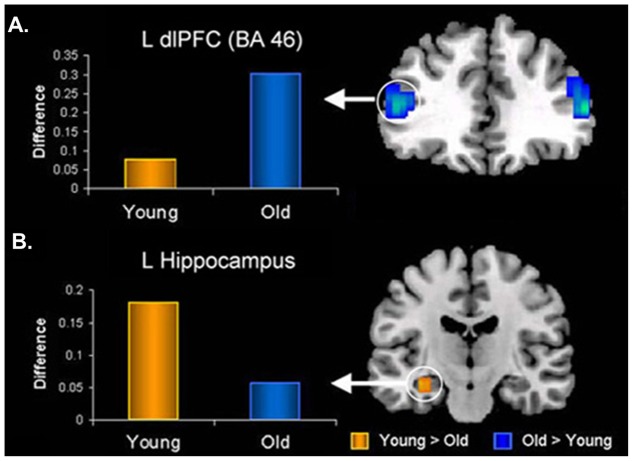
Opposing age-related changes in the PFC and MTL connectivity with the AMY during emotional memory encoding. **(A)** Age-related enhanced functional connectivity between AMY and dorsolateral PFC (dlPFC). **(B)** Age-related decreased functional connectivity between AMY and hippocampus (HC) during encoding of memories for negative pictures. L, left; BA, Brodmann Area. Adapted from St. Jacques et al. (2009).

In the memory domain, many studies have provided evidence supporting the positivity effect observed in healthy older adults, as reflected in their tendency to remember more positive and fewer negative memories for personal or laboratory events (e.g., Charles et al., 2003; Kennedy et al., 2004; Comblain et al., 2005; Schlagman et al., 2006; Fernandes et al., 2008; Spaniol et al., 2008; Ebner and Johnson, 2009; St. Jacques et al., 2009). At the neural level, the AMY-MTL and PFC have been highlighted as key regions subserving the memory-enhancing effect of emotion in aging, with the PFC mechanisms playing a crucial role (reviewed in Dolcos et al., 2012). For example, Murty et al. (2009) and St. Jacques et al. (2009) both identified reduced connectivity between the AMY and HC, paired with enhanced connectivity between the AMY and lateral PFC, associated with successful encoding of negative information in older compared to younger adults (**Figure 7**). Additionally, recent investigations of effective connectivity during emotional encoding identified both (1) greater positive modulation of MTL engagement by PFC, and (2) greater *intra*-regional connectivity within PFC (e.g., orbital, medial, and lateral), during the encoding of positive information in older than in younger adults (Addis et al., 2010; Waring et al., 2013). Finally, older adults who showed greater connectivity between AMY and mPFC *at rest* also showed (1) increased mPFC engagement during the encoding of emotional faces, and (2) a greater positivity effect in a subsequent memory recognition test (i.e., more likely to remember positive as opposed to negative faces), whereas these patterns were not shown in younger adults (Sakaki et al., 2013). Taken together, these findings emphasize an age-related strengthening in AMY-PFC connectivity that is overall linked to enhanced emotional memory encoding, and also associated with the positivity effect observed in older adults. Importantly, age-related decrease in the engagement of the AMY-MTL mechanisms coupled with the increased involvement of the AMY-PFC mechanisms in emotional memory is also consistent with the PASA (Posterior–Anterior Shift in Aging) model in the cognitive aging literature, characterized by reduced contribution of the posterior brain regions compensated by greater recruitment of the PFC regions (Davis et al., 2008).

In addition, extant behavioral findings suggest that older adults have greater susceptibility compared to young adults to the aforementioned memory trade-off effect between emotional/central vs. non-emotional/peripheral aspects of an event (Kensinger et al., 2005, 2007b; Nashiro and Mather, 2011). These age-related differences have been associated with older adults’ relatively decreased tendency to engage in particular encoding strategies (e.g., broad allocation of attention to contextual features of stimuli) that help younger adults reduce the negative influence of the trade-off effect. This suggests that older adults may have particular difficulty in disengaging attention from emotionally salient features of stimuli (Kensinger et al., 2005, 2007b). Furthermore, there is evidence that emotional arousal enhances memory for information about intrinsically linked contextual aspects (e.g., stimuli and their location) through memory binding in younger adults but not in older adults, thus suggesting that possibly limited cognitive resources in older adults may lead them to remember only the gist but not the associated details (Nashiro and Mather, 2011). This evidence is consistent with previous research documenting age-related impairments in inhibition and attentional control operations (reviewed in Fabiani, 2012), and various forms of associative memory (Old and Naveh-Benjamin, 2008). Finally, although evidence regarding the underlying mechanisms of age-related deficits in associative memory is currently limited, a recent investigation of the effect of age on memory processes found a significant link between associative memory performance and HC volume in older adults, consistent with the idea that this region is involved in representations of item-context relations (Henson et al., 2016).

## Summary and Future Directions

**FIGURE 8:**
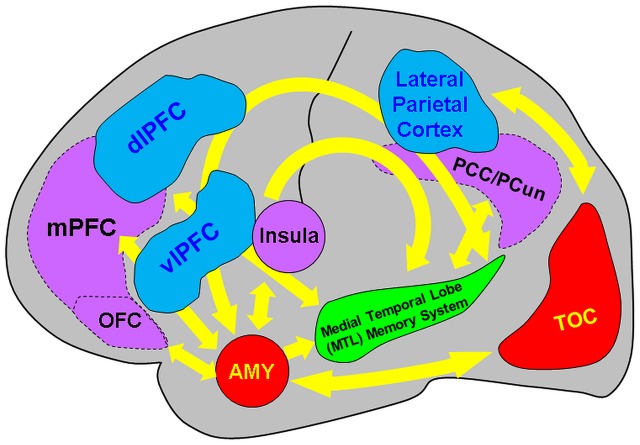
Diagram summarizing the neural correlates of the memory-enhancing effect of emotion, as resulted from brain imaging studies. Two main basic mechanisms involved in the memory-enhancing effect of emotion were identified: (I) one based in the MTL (AMY and MTL Memory System = HC and associated parahippocampal cortices) and (II) the other also involving non-MTL regions, such as the medial and dorso/ventrolateral prefrontal cortex (mPFC and dlPFC/vlPFC, respectively), among others (e.g., parietal cortex). The AMY and the MTL memory regions interact through direct/automatic neurohormonal mechanisms that contribute to the memory-enhancing effect of emotion (bottom-up mechanism), whereas PFC is part of a mechanism that has an indirect/mediated involvement in emotional memories, by enhancing strategic, semantic, working memory, and attentional processes (top-down mechanism). In addition to basic effects of emotion, encoding of positive and negative emotions is also associated with relatively increased involvement of the PFC and temporo-occipital cortices (TOC), respectively. Moreover, investigation of social emotional memory identified valence-specific engagement of other brain regions that contribute to enhanced emotional memories in social contexts – i.e., memory for socially relevant information involves activity in and interactions between the medial orbitofrontal cortex (OFC) and the MTL in the case of items with positive connotations and the Insula and the MTL for items with negative connotations. Moreover, the midline cortical regions of the default mode network [mPFC, posterior cingulate cortex/precuneus (PCC/PCun)] are also involved in encoding of self-relevant information in social context. In addition, investigation of the impact of emotion regulation on emotional memory identified bidirectional relations between the MTL and the PFC regions associated with specific emotion regulation strategies, involving (i) the lateral/medial PFC in top-down modulation of the AMY-MTL mechanisms in emotional memory encoding and retrieval, and (ii) the AMY signaling medial PFC the need to exert control over emotional stimuli, resulting in overall reduced emotional experience, during autobiographical retrieval. Both the bottom-up and top-down mechanisms involved in emotional episodic memory are modulated by individual differences related to personality, sex, and age. Adapted from Dolcos et al. (2011, 2012) and Denkova et al. (2015).

The present review discussed findings from brain imaging studies investigating the neural correlates of encoding and retrieving emotional memories, and how they are modulated by factors linked to *social information*, *emotion regulation*, *associative/relational memory*, and *individual differences* in personality traits, sex, and age. The available evidence points to the involvement of and interaction between direct/bottom-up MTL-based and indirect/top-down PFC-based mechanisms associated with the impact of emotion on memory, which seem to be modulated by basic affective properties (arousal and valence) and by the four factors discussed here (see **Figure 8** and **Table 1**).

**Table 1:** Summary of main functions of the PFC, AMY, and HC in emotional memory encoding and retrieval across four domains.

		Social emotional memory	Emotion regulation	Relational memory	Individual differences
**PFC**		Encoding of stimuli with self- and social relevance (mPFC, OFC), especially positive ones (**w/ AMY, HC**).	Enhanced (by reappraisal) vs. impaired (by expressive suppression) encoding of stimuli (latPFC, dmPFC) (**w/ HC**).	Retrieval of stimuli encoded in association with emotional information (**w/ AMY, HC**).	*Sex* : Increased activity linked to emotional autobiographical memory recollection in women vs. men (latPFC).
		Retrieval of stimuli previously learned in a socially emotional context.	Impaired (by thought suppression) retrieval of stimuli (latPFC) (**w/ HC**).		*Age* : Increased functional connectivity (**w/ AMY**) linked to emotional encoding and retrieval (e.g., positivity effect) in older vs. younger adults.
			Reduced (by focused attention) experience of emotions associated with recollection of emotional autobiographical memories (vmPFC) (**w/ AMY**).		

**MTL**	**AMY**	Encoding of stimuli with self- and social relevance.	Enhanced (by reappraisal) encoding of stimuli (**w/ HC, latPFC**).	Encoding of within-object, intrinsically-related, or “unitized” features related to emotional stimuli (**w/ memory-related MTL regions**).	*Personality* : Enhanced encoding of positive (by extraversion) vs. negative (by neuroticism and anxiety) stimuli.
		Encoding of socially positive (**w/ OFC**) and negative (**w/ Insula**) stimuli.	Reduced (by focused attention) experience of emotions associated with recollection of emotional autobiographical memories (**w/ vmPFC**).	Retrieval of stimuli encoded in association with emotional information (**w/ HC**).	*Sex* : Enhanced encoding and consolidation in women (left) and in men (right); enhanced recollection of negative autobiographical memories in women vs. men.
		Retrieval of stimuli previously learned in a socially emotional context.			*Age* : Decreased (**w/ HC**) and increased (**w/ latPFC**) functional connectivity linked to negative memory encoding in older vs. younger adults.

	**HC**	Encoding of self-relevant stimuli (**w/ mPFC**, **Precuneus**).	Enhanced (by reappraisal) vs. impaired (by expressive suppression) encoding of stimuli (**w/ AMY, latPFC**).	Encoding of relational (e.g., item-context) memory representations (**w/ MTL**).	*Personality* : Enhanced learning of negative associations (by neuroticism).
		Encoding of socially positive (**w/ OFC**) and negative (**w/ Insula**) stimuli (also **w/ AMY, FFA**).	Impaired (by thought suppression) retrieval of stimuli (**w/ latPFC**).	Retrieval of stimuli encoded in association with emotional information (**w/ AMY**).	*Age* : Decreased (**w/ AMY**) functional connectivity linked to negative memory encoding in older vs. younger adults.
		Retrieval of stimuli previously learned in a socially emotional context.			


*The table identifies the main functions of the prefrontal cortex (PFC) as well as the medial temporal lobe (MTL) structures [amygdala (AMY), hippocampus (HC)] related to the four research domains discussed in the present review. Regions in **bold** font have been identified as showing changes in functional connectivity linked to the particular function being discussed. OFC, orbitofrontal cortex; (d/v)mPFC, (dorso-/ventro-) medial prefrontal cortex; latPFC, lateral prefrontal cortex; FFA, fusiform face area.*

Despite a rapidly growing body of literature elucidating the mechanisms underlying emotional memory in the context of the four emerging directions discussed in this review, a number of issues remain unclear. Regarding the role of *social information*, one issue that has been relatively less explored in the literature concerns the mechanisms underlying the impact of emotion control/regulation on memory processes in social context. This is surprising, given the evidence that the ability to control and experience emotions appropriately is essential in successful social interactions (Gross, 2002). Available behavioral evidence shows that engaging reappraisal and suppression during social interactions is associated with enhanced memory for conversation content vs. emotional reactions in the context of discussing relationship conflicts, respectively (Richards et al., 2003). More recently, it has been shown that social regulation (e.g., holding someone’s hand during an emotional experience) can lead to decreased memory for negative images (Flores and Berenbaum, 2017). Complementing these findings, recent neuroimaging evidence suggests that regulation of emotions experienced through interaction with others may be subserved by dissociable neural networks, compared to those involved in regulating emotions experienced individually (Grecucci et al., 2015). Thus, clarification of the mechanisms involved in regulating how we experience and interpret our own emotions and those of others, and how these processes influence our remembrance of social experience, may allow us to better understand the complex nature of human social interactions.

Regarding the role of *emotion regulation* on emotional memory, further investigations are needed to clarify the impact of various ER strategies that can be used to cope with emotional memories. Given that emerging research highlights the effectiveness of attentional deployment strategies (distraction, FA) (Denkova et al., 2015), future investigations of the impact of ER on memory should include more elaborated practices and training that rely on attentional control. Mindfulness, which involves attention to the present moment without emotional reactivity and elaboration, is such a technique that could be used as a training method to reduce the impact of distressing emotional memories. This line of future research seems promising, since there is preliminary evidence showing that mindfulness training, which has been associated with increased well-being, can impact the neural correlates of emotion processing (Allen et al., 2012; Desbordes et al., 2012) and increase the specificity of AMs in healthy (Heeren et al., 2009) and formerly depressed (Williams et al., 2000) individuals.

Regarding *relational emotional memory*, the role of stress on neural memory binding has been less explored. Recent lines of research (e.g., Joëls et al., 2011; van Ast et al., 2013; Hermans et al., 2014; Vogel et al., 2016) emphasize two different corticosteroid actions critical for memory processes. On the one hand, to promote survival, shortly after stress, non-genomic corticosteroids interact with noradrenaline to enable immediate fight-flight responses and to focus attention on gist-based information at the cost of remembering contextual details. On the other hand, some hours after stress, slower long-lasting, gene-mediated, corticosteroid actions are thought to facilitate restorative processes, involving enhanced executive control (Hermans et al., 2014) and remembering events in a more contextual manner (van Ast et al., 2013). Future studies investigating opposing effects of emotion on associative binding might consider these time-dependent stress actions (see also Dolcos, 2014).

Finally, regarding the role of *individual differences*, whereas investigation of individual differences in emotion-related domains seems promising to explain differences in the heightened sensitivity to and enhanced encoding of emotional information (e.g., Haas and Canli, 2008), relatively less is known about the impact of individual differences in the cognitive domain. The latter may be of particular importance regarding the implementation and impact of ER strategies during memory formation. For instance, there is evidence showing that higher working memory capacity is associated with better suppression of negative emotions (Schmeichel et al., 2008), and that working memory training may lead to better emotion control through enhanced efficiency of the fronto-parietal network (Schweizer et al., 2013). Hence, this seems a promising research avenue. Moreover, recent studies also highlight the importance of taking into account individual differences in multiple domains concomitantly, given the highly interactive nature of their effects on these phenomena. For example, as discussed above, neuroticism but not extraversion was linked to sex differences in the recollection of emotional memories and the emotional states post-memory retrieval (Denkova et al., 2012). Also, neuroticism at older ages is linked to greater levels of negative affect (Mroczek et al., 2006) and an elevated risk of developing mild cognitive impairment (Wilson et al., 2005). At the neural level, neuroticism has been shown to interact with both sex and age in influencing the volume of brain regions associated with emotion processing (e.g., AMY, fusiform gyrus) (Jackson et al., 2011; Nostro et al., 2016). Although current research is still limited, available evidence shows that taking into consideration multiple domains of individual differences will be crucial in gaining a comprehensive understanding of the mechanisms involved in emotional memory. Hence, examining the interacting influences of multiple individual difference factors will be a key approach in better understanding how individual variations may impact the mechanisms underlying emotional memory.

## Author Contributions

The authors’ main contributions to specific sections are as follows: FD contributed to all sections. YK and SD contributed to the sections on social emotional memory, the role of emotion regulation, and the role of individual differences in emotional memory. MW contributed to the section on the impact of emotion on relational memory. MM contributed to the section on the role of individual differences, and TT contributed to the section on the role of emotion regulation in the impact of emotion on memory.

## Conflict of Interest Statement

The authors declare that the research was conducted in the absence of any commercial or financial relationships that could be construed as a potential conflict of interest.

## Footnotes

1 Although not the focus of this review, it should be noted that there has been recent seminal work using intracranial recordings highlighting the role of brain regions such as the anterior thalamic nucleus (ATN) in support of successful memory encoding (Sweeney-Reed et al., 2014, 2015). More specifically, recent evidence shows that neocortical-ATN theta oscillatory phase synchrony, neocortical-theta-to-ATN-gamma cross-frequency coupling, and phase alignment in the theta rhythm predict successful encoding, suggesting an active role of the ATN during memory formation (Sweeney-Reed et al., 2014, 2015).

2 It is important to note that in addition to the emerging aspects discussed here, understanding how the mechanisms underlying emotional memory are altered in clinical conditions is also important. Clarification of the neural mechanisms involved in emotional memory, including the aspects highlighted in this review, allows for better understanding of alterations in the neural mechanisms of dysfunctional emotional memory in clinical conditions, such as affective disturbances (anxiety, depression, stress-related, etc.). Thus, it is crucial to understand the contribution of emotion- and memory-related brain regions, and their interaction with cognitive control regions, to emotional memory. Although an elaborated discussion of these issues is not possible due to space limitations, we briefly illustrate how clinical alterations in all four domains discussed here can be identified in PTSD. For example, there is evidence that PTSD is associated with impaired social cognitive processing, such as reduced social problem solving (Sutherland and Bryant, 2008) and interpersonal functioning (Sharp et al., 2012), and with emotion regulation difficulties (Ehring and Quack, 2010) (see also Rauch et al., 2006; Shin and Liberzon, 2009; Brown and Morey, 2012; Hayes et al., 2012). Additionally, associative memory mechanisms are also compromised in PTSD patients, who tend to have gist-based or decontextualized distressing memories (McNally et al., 1994; Kaspi et al., 1995; Harvey et al., 1998; Hayes et al., 2011). It has been proposed that impaired ability to recollect contextual details associated with the traumatic events is due to hyperarousal during encoding, which leads to functional disorganization of the memory-related AMY-HC mechanisms (Dolcos, 2013). Finally, individual differences such as neuroticism have been shown to be associated with increased relative risk of PTSD in response to trauma (Breslau and Schultz, 2013). Thus, investigation of emotional memory in relation to the various domains highlighted in this review has the potential to contribute to our better understanding of the neural mechanisms of altered emotional memory in affective disturbances, and of identifying potential therapeutic interventions in these and other related clinical conditions.
